# Discrepancies in intensive care unit triage decisions for patients with advanced cancer: a Brazilian survey of intensivists and oncologists

**DOI:** 10.62675/2965-2774.20260204

**Published:** 2026-01-14

**Authors:** Carla Marchini Dias da Silva, Beatriz Araújo

**Affiliations:** 1 Intensive Care Unit, A.C. Camargo Cancer Center São Paulo SP Brazil Intensive Care Unit, A.C. Camargo Cancer Center - São Paulo (SP), Brazil.; 2 Universidade Nove de Julho Department of Medicine São Paulo SP Brazil Department of Medicine, Universidade Nove de Julho - São Paulo (SP), Brazil.

Oncologists and intensivists often face clinical and ethical challenges when determining goals of care and treatment options for critically ill patients with cancer.^(1)^ When intensive care offers no realistic chance of clinical improvement, its use may become non-beneficial, raising concerns about disproportionate care, resource allocation, and patient suffering.^(2–4)^ While previous studies have shown that oncologists and intensivists may adopt distinct approaches in managing cancer patients during intensive care unit (ICU) stays,^(5)^ few have investigated whether these specialties also diverge in their triage criteria, particularly in the Brazilian context. Understanding how each specialty perceives the potential benefits and limitations of intensive care could facilitate more aligned decision-making, promote goal-concordant care, and help reduce interprofessional conflict. In this study, we evaluated the consistency of ICU triage decisions among Brazilian intensivists and oncologists/hematologists, based on the prioritization framework proposed by the Society of Critical Care Medicine (SCCM).^(6)^

A national cross-sectional electronic survey was conducted among intensivists, oncologists, and hematologists. Participants evaluated ten clinical vignettes based on real cases and classified each scenario using SCCM ICU priority levels (1 = highest, 5 = inappropriate), alongside predictions for ICU, hospital, and 1-year survival (Research form - Supplementary Material). Fleiss’ kappa coefficient was used to assess inter-rater agreement. Sociodemographic and professional characteristics were also collected.

Of 432 physicians invited, 111 responded (25.7%): 90 intensivists, 17 oncologists, and 4 hematologists (Table 1). The overall level of agreement was low (κ = 0.215; 95%CI 0.208 - 0.221), including that between the two specialties (Figure 1).

**Table 1:** Characteristics of participating physicians

Characteristics			Intensivists (n = 90)	Oncologists/hematologists (n = 21)	p value
Female			46 (51.1)	11 (52.4)	0.92
Age range (years)					0.13
	20 - 30		7 (7.8)	5 (23.8)	
	31 - 40		45 (50.0)	7 (33.3)	
	41 - 50		27 (30.0)	7 (33.3)	
		Over 51 years	11 (12.2)	2 (9.6)	
Married/in a stable union			65 (72.2)	14 (66.7)	0.76
Atheist			16 (17.8)	2 (9.5)	0.20
Palliative care training during medical education			72 (80.0)	14 (66.7)	0.19
Articles or lectures on palliative/end-of-life care in the past year			86 (95.6)	18 (85.7)	0.09
Professional characteristics of intensivists (n = 90)					
With board certification or medical residency			82 (91.1)		
Works exclusively in a dedicated oncology ICU			12 (13.5)		
Works exclusively in the general ICU			24 (27)		
Works exclusively in public hospital ICUs			8 (9)		
Works exclusively in private hospital ICUs			34 (38.2)		

ICU - intensive care unit. Results expressed as n (%).

**Figure 1:**
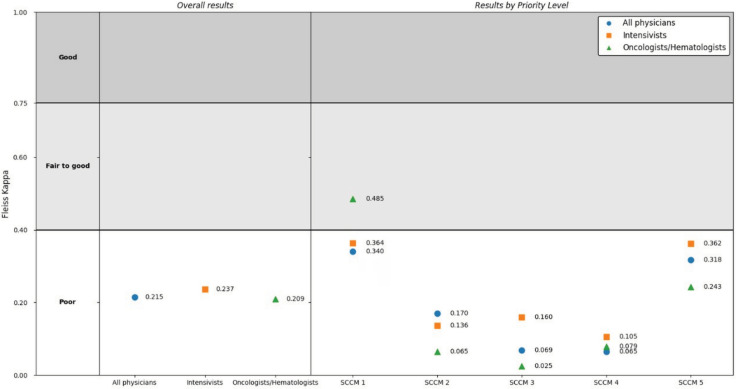
Fleiss’ kappa coefficient for the Society of Critical Care Medicine prioritization model according to medical specialty.

SCCM - Society of Critical Care Medicine.

The most significant divergence occurred in two vignettes involving end-of-life scenarios (Supplementary Material). In vignette 8, a patient with advanced dementia (Eastern Cooperative Oncology Group scale [ECOG] 4), 40.3% of intensivists *versus* 11.1% of oncologists/hematologists deemed ICU admission inappropriate (priority 5; p = 0.048). In vignette 10, involving a patient under best supportive care for metastatic breast cancer, 47.8% of intensivists *versus* 22.2% of oncologists/hematologists selected priority 5 (p = 0.039). Divergent prognostic expectations were also observed in 1-year survival predictions in vignettes 1 and 5 (p = 0.006 and 0.002, respectively).

No demographic or professional factors (e.g., age, religion, palliative care training) were associated with higher agreement (Table 1S - Supplementary Material). These findings echo earlier studies that reveal that ICU admission decisions are highly influenced by subjective judgments and psychological traits, such as optimism, mortality aversion, discomfort with delivering bad news, and the degree of physician emotional involvement.^(7–9)^

Our findings indicate a potential variability in triage decisions and prognostic prediction, reflecting differing perspectives: intensivists focus on acute organ dysfunction and short-term functional outcomes, while oncologists emphasize disease trajectory and therapeutic potential.^(5)^ A longstanding doctor-patient relationship may reduce prognostic accuracy,^(10)^ and oncologists are more likely to overestimate survival compared to other specialists, potentially contributing to this divergence.^(10,11)^

This study has several limitations. First, the low response rate (25.7%), especially among oncologists and hematologists, may introduce selection bias and limit generalizability. Second, the vignette-based methodology may not fully capture the complexity of real-life decision-making. Third, social desirability bias may have influenced some responses. Fourth, data on geographic location, institutional characteristics (especially among oncologists/hematologists), and participants’ years of experience were incomplete or unavailable. Fifth, the study did not include methodological triangulation. Finally, reliance on a single prioritization model (SCCM) may not fully reflect all relevant factors involved in ICU admission decisions for critically ill patients with cancer.

In conclusion, ICU triage for patients with advanced cancer remains inconsistent. Implementing oncology-specific guidelines and fostering interdisciplinary decision-making are key to ensuring appropriate and compassionate care.^(12)^ Enhanced training in palliative care and prognostic communication can further reduce non-beneficial interventions and align treatments with patient values.^(2,13)^

## ETHICS APPROVAL

The study was approved by the Research Ethics Committee of A.C. Camargo Cancer Center (CAAE: 77874224.4.0000.5432), and all participants provided formal consent via the Informed Consent Form.

## Data Availability

The datasets used and analyzed in our study are available from the corresponding author upon reasonable request.
