# Supplementary material for: Discrepancies in intensive care unit triage decisions for patients with advanced cancer: a Brazilian survey of intensivists and oncologists
Source: Crit Care Sci. 2026 Jan 14;38:e20260204. doi: 10.62675/2965-2774.20260204 (PMC12977219; doi:10.62675/2965-2774.20260204)
Supplement: Supplementary Material [file 2965-2774-ccsci-38-e20260204-suppl1.pdf]

Critical Care Science
https://doi.org/10.62675/2965-2774.20260204

SUPPLEMENTARY MATERIAL

Discrepancies in intensive care unit triage decisions for patients with advanced cancer: a Brazilian survey of intensivists and oncologists

Carla Marchini Dias da Silva[1], Beatriz Araújo[2]

RESEARCH FORM

Part I: Demographic information

1. Gender
- ( ) Female
- ( ) Male
- ( ) Prefer no to disclose

2. Age
- ( ) Between 20 and 30 years
- ( ) Between 31 and 40 years
- ( ) Between 41 and 50 years
- ( ) Between 51 and 60 years
- ( ) Over 60 years

3. Marital status
- ( ) Single_______
- ( ) In a stable relationship
- ( ) Married
- ( ) Widowed
- ( ) Other: ___________________

This is an open access article under the CC BY license (https://creativecommons.org/licenses/by/4.0/).

4. Religion/belief

( ) Catholic

( ) Evangelical

( ) Spiritist

( ) Jewish

( ) Buddhist

( ) Islamic

( ) Umbanda, Candomblé, or other Afro-Brazilian religions

( ) Agnostic

( ) Atheist

( ) Other: ____________________

5. Medical specialty

( ) Critical care medicine

( ) Oncology

( ) Hematology

( ) Other: ____________________

6. Have you received palliative care training during medical school, residency, or postgraduate studies?

( ) Yes

( ) No

7. In the past year, have you read articles on end-of-life care or palliative care, attended conferences, or participated in lectures on the topic?

( ) Yes

( ) No

If you work in Critical Care Medicine:

8. Do you have a completed residency, or a specialist title recognized by Associação de Medicina Intensiva Brasileira (AMIB)?

( ) Yes

( ) No

9. Work setting:

( ) Exclusively in an oncology hospital

( ) Exclusively in a general hospital

( ) Both

10. Intensive care unit (ICU) type where you work:

( ) Public ICU

( ) Private ICU

( ) Both

Part II: Patient classification and prognosis based on Society of Critical Care Medicine Guidelines

Please classify the patient from the clinical cases provided below, using your clinical judgment, in accordance with the priority categories outlined by the Society of Critical Care Medicine (SCCM), which prioritizes patients for ICU admission based on their likelihood of survival and recovery. Subsequently, predict the patient's outcome for each vignette, considering the probability of survival.

1) LGC, 73 years old, ECOG 1, diagnosed with mucinous adenocarcinoma of the rectosigmoid with KRAS mutation in December 2023. Currently presenting peritoneal metastases. Underwent rectosigmoidectomy and lymphadenectomy in March 2024, complicated by anastomotic dehiscence, requiring drainage of a collection and prolonged antibiotic therapy. Awaiting clinical improvement to initiate first-line treatment. Admitted to the emergency department with fever, nausea, vomiting, and intense abdominal pain. Blood pressure: 70/65mmHg, with no improvement after fluid resuscitation with 1500mL of crystalloids.

( ) Priority 1: Critically ill patients requiring therapies or monitoring that can only be provided in the ICU.

( ) Priority 2: Similar patients as above, but with significantly lower chances of recovery due to acute illness or underlying disease.

( ) Priority 3: Patients with organ dysfunction needing therapies or monitoring that could be provided outside the ICU.

( ) Priority 4: Similar patients as above, but with significantly lower chances of recovery due to acute illness or underlying disease.

( ) Priority 5: Terminal or moribund patients with little to no chance of recovery. These patients are generally not appropriate for ICU admission and should preferably receive palliative care.

Patient outcome prediction:

In the ICU:

( ) Discharged alive

( ) Death

In the hospital:

( ) Discharged alive

( ) Death

In 1 year:

( ) Alive

( ) Dead

2) **A 53-year-old patient with lymphoblastic leukemia/lymphoma, electively admitted to a ward for allogeneic bone marrow transplantation. On Day +4, she develops fever, blood pressure of 90/60mmHg, tachypnea with respiratory rate of 32 bpm showing signs of respiratory distress, oxygen saturation of 83% on room air, and bilateral crackles up to the lung apices. Also presents with cough producing bloody sputum and pancytopenia (platelets: 5,000).**

( ) Priority 1: Critically ill patients requiring therapies or monitoring that can only be provided in the ICU.

( ) Priority 2: Similar patients as above, but with significantly lower chances of recovery due to acute illness or underlying disease.

( ) Priority 3: Patients with organ dysfunction needing therapies or monitoring that could be provided outside the ICU.

( ) Priority 4: Similar patients as above, but with significantly lower chances of recovery due to acute illness or underlying disease.

( ) Priority 5: Terminal or moribund patients with little to no chance of recovery. These patients are generally not appropriate for ICU admission and should preferably receive palliative care.

Patient outcome prediction:

In the ICU:

( ) Discharged alive

( ) Death

In the hospital:

( ) Discharged alive

( ) Death

In 1 year:

( ) Alive

( ) Dead

3) **LFT, 66 years old, diagnosed with right breast cancer – JQL – IDC cT2cN0. Late postoperative period after mastectomy + lymph node dissection. Oxygen-dependent chronic obstructive pulmonary disease (COPD) patient with multiple exacerbations in the past year, ECOG 3. Admitted to the ward due to another COPD exacerbation. After 48 hours of antibiotic and corticosteroid therapy, she develops respiratory discomfort, dyspnea, desaturation, and decreased level of consciousness.**

( ) Priority 1: Critically ill patients requiring therapies or monitoring that can only be provided in the ICU.

( ) Priority 2: Similar patients as above, but with significantly lower chances of recovery due to acute illness or underlying disease.

( ) Priority 3: Patients with organ dysfunction needing therapies or monitoring that could be provided outside the ICU.

( ) Priority 4: Similar patients as above, but with significantly lower chances of recovery due to acute illness or underlying disease.

( ) Priority 5: Terminal or moribund patients with little to no chance of recovery. These patients are generally not appropriate for ICU admission and should preferably receive palliative care.

Patient outcome prediction:

In the ICU:

( ) Discharged alive

( ) Death

In the hospital:

( ) Discharged alive

( ) Death

In 1 year:

( ) Alive

( ) Dead

4) RVS, 38 years old, diagnosed with squamous cell carcinoma of the tongue, operated in Feb/22, with adjuvant radiotherapy and chemotherapy. Local and pulmonary recurrence in Apr/23. New pulmonary disease progression in Jan/24. Currently receiving second-line treatment with Nivolumab (Cycle 2 on 03/16). Admitted to the emergency department with cough, fever, and signs of respiratory distress. Oxygen saturation of 76% on room air and blood pressure of 70/40mmHg. Patient is cachectic, ECOG 3, with a history of recent hospitalizations due to infectious decompensation.

( ) Priority 1: Critically ill patients requiring therapies or monitoring that can only be provided in the ICU.

( ) Priority 2: Similar patients as above, but with significantly lower chances of recovery due to acute illness or underlying disease.

( ) Priority 3: Patients with organ dysfunction needing therapies or monitoring that could be provided outside the ICU.

( ) Priority 4: Similar patients as above, but with significantly lower chances of recovery due to acute illness or underlying disease.

( ) Priority 5: Terminal or moribund patients with little to no chance of recovery. These patients are generally not appropriate for ICU admission and should preferably receive palliative care.

Patient outcome prediction:

In the ICU:

( ) Discharged alive

( ) Death

In the hospital:

( ) Discharged alive

( ) Death

In 1 year:

( ) Alive

( ) Dead

5) **HVL, 24 years old, ECOG 1, with testicular mass and metastases in the liver, lungs, and retroperitoneum, as well as extensive thrombosis in the inferior vena cava extending to the renal veins and distention of the small intestine; still without a definitive histopathological diagnosis (suspected testicular germ cell tumor). Admitted to the ward for diagnostic investigation and initiation of treatment. He develops nausea, vomiting, hypokalemia, and high-rate atrial fibrillation (HR 165 bpm), BP 100/50mmHg, capillary refill < 3s, without dyspnea or chest pain.**

( ) Priority 1: Critically ill patients requiring therapies or monitoring that can only be provided in the ICU.

( ) Priority 2: Similar patients as above, but with significantly lower chances of recovery due to acute illness or underlying disease.

( ) Priority 3: Patients with organ dysfunction needing therapies or monitoring that could be provided outside the ICU.

( ) Priority 4: Similar patients as above, but with significantly lower chances of recovery due to acute illness or underlying disease.

( ) Priority 5: Terminal or moribund patients with little to no chance of recovery. These patients are generally not appropriate for ICU admission and should preferably receive palliative care.

Patient outcome prediction:

In the ICU:

( ) Discharged alive

( ) Death

In the hospital:

( ) Discharged alive

( ) Death

In 1 year:

( ) Alive

( ) Dead

6) **BGL, 69 years old, diagnosed with multiple myeloma, IgG Lambda (ISS III, Durie and Salmon IIIB), ECOG 2. Patient with CKD not on dialysis, insulin-dependent diabetes, CAD (post-myocardial revascularization in 2011, drug-eluting stent in 2021, new coronary angiography on 19/12/22 with stent). Presenting to the ER with persistent chest pain for 24 hours, without associated symptoms. Admission ECG shows no ST-segment changes, and the first troponin is negative. Patient remains hemodynamically stable.**

( ) Priority 1: Critically ill patients requiring therapies or monitoring that can only be provided in the ICU.

( ) Priority 2: Similar patients as above, but with significantly lower chances of recovery due to acute illness or underlying disease.

( ) Priority 3: Patients with organ dysfunction needing therapies or monitoring that could be provided outside the ICU.

( ) Priority 4: Similar patients as above, but with significantly lower chances of recovery due to acute illness or underlying disease.

( ) Priority 5: Terminal or moribund patients with little to no chance of recovery. These patients are generally not appropriate for ICU admission and should preferably receive palliative care.

Patient outcome prediction:

In the ICU:

( ) Discharged alive

( ) Death

In the hospital:

( ) Discharged alive

( ) Death

In 1 year:

( ) Alive

( ) Dead

7) **ADF, 80 years old, diagnosed with metastatic melanoma (thorax, lungs, lymph nodes, CNS), post-axillary lymphadenectomy in 2014. Received Nivo + Ipi; Nivo + RDT; disease progression despite treatment. Admitted on 07/04/24 with a diagnosis of dengue. Hospitalized in the ward for platelet count control, later developing drowsiness, fever, and 89% saturation on room air, with no signs of respiratory distress. Personal history includes COPD (not requiring O2), atrial arrhythmia, dementia syndrome partially dependent for activities of daily living, and active alcohol use.**

( ) Priority 1: Critically ill patients requiring therapies or monitoring that can only be provided in the ICU.

( ) Priority 2: Similar patients as above, but with significantly lower chances of recovery due to acute illness or underlying disease.

( ) Priority 3: Patients with organ dysfunction needing therapies or monitoring that could be provided outside the ICU.

( ) Priority 4: Similar patients as above, but with significantly lower chances of recovery due to acute illness or underlying disease.

( ) Priority 5: Terminal or moribund patients with little to no chance of recovery. These patients are generally not appropriate for ICU admission and should preferably receive palliative care.

Patient outcome prediction:

In the ICU:

( ) Discharged alive

( ) Death

In the hospital:

( ) Discharged alive

( ) Death

In 1 year:

( ) Alive

( ) Dead

8) **AFM, 75 years old, diagnosed with mature low-grade B-cell lymphoma. Patient with advanced Parkinson's disease, ECOG 4, hospitalized in the ward for completion of pneumonia treatment. Presents with hyperactive** delirium **and difficult-to-manage aggression. Afebrile for 48 hours, with progressive improvement in laboratory results and stable vital signs.**

( ) Priority 1: Critically ill patients requiring therapies or monitoring that can only be provided in the ICU.

( ) Priority 2: Similar patients as above, but with significantly lower chances of recovery due to acute illness or underlying disease.

( ) Priority 3: Patients with organ dysfunction needing therapies or monitoring that could be provided outside the ICU.

( ) Priority 4: Similar patients as above, but with significantly lower chances of recovery due to acute illness or underlying disease.

( ) Priority 5: Terminal or moribund patients with little to no chance of recovery. These patients are generally not appropriate for ICU admission and should preferably receive palliative care.

Patient outcome prediction:

In the ICU:

( ) Discharged alive

( ) Death

In the hospital:

( ) Discharged alive

( ) Death

In 1 year:

( ) Alive

( ) Dead

9) **ASC, 42 years old, diagnosed with metastatic anal canal SCC (spread to CNS, lymph nodes, and pelvis), previously treated with multiple therapies, and HIV infection. Discharged from this hospital on 14/04/24 after acute renal failure, reversed with double-J stent replacement. Two days later, develops progressive vomiting, fatigue, malaise, lower abdominal discomfort, and cessation of stool elimination via colostomy, associated with distension of the collection bag and significant protrusion of bowel loops. Presented to the ER with BP 80/40mmHg and temperature 38°C. In the prior hospitalization, the family and patient were informed about a reserved prognosis and opted for prioritizing comfort in case of clinical deterioration.**

( ) Priority 1: Critically ill patients requiring therapies or monitoring that can only be provided in the ICU.

( ) Priority 2: Similar patients as above, but with significantly lower chances of recovery due to acute illness or underlying disease.

( ) Priority 3: Patients with organ dysfunction needing therapies or monitoring that could be provided outside the ICU.

( ) Priority 4: Similar patients as above, but with significantly lower chances of recovery due to acute illness or underlying disease.

( ) Priority 5: Terminal or moribund patients with little to no chance of recovery. These patients are generally not appropriate for ICU admission and should preferably receive palliative care.

Patient outcome prediction:

In the ICU:

( ) Discharged alive

( ) Death

In the hospital:

( ) Discharged alive

( ) Death

In 1 year:

( ) Alive

( ) Dead

10) ACG, 55 years old, diagnosed with left breast IDC, luminal B, HER2-negative metastatic to bone and liver, previously treated with multiple therapies, currently receiving best supportive care (BSC). Presents to the ER with complaints of nausea, vomiting, back pain, fatigue, and increased abdominal volume. Decided to be admitted to the ward for paracentesis and symptom management. One day later, develops erythema, redness, and pain at the puncture site, associated with hypotension (BP 70/50mmHg) and tachycardia (HR 132 bpm).

( ) Priority 1: Critically ill patients requiring therapies or monitoring that can only be provided in the ICU.

( ) Priority 2: Similar patients as above, but with significantly lower chances of recovery due to acute illness or underlying disease.

( ) Priority 3: Patients with organ dysfunction needing therapies or monitoring that could be provided outside the ICU.

( ) Priority 4: Similar patients as above, but with significantly lower chances of recovery due to acute illness or underlying disease.

( ) Priority 5: Terminal or moribund patients with little to no chance of recovery. These patients are generally not appropriate for ICU admission and should preferably receive palliative care.

Patient outcome prediction:

In the ICU:

( ) Discharged alive

( ) Death

In the hospital:

( ) Discharged alive

( ) Death

In 1 year:

( ) Alive

( ) Dead

Table 1S - Fleiss' Kappa coefficient according to participant characteristics

Characteristics	Fleiss' Kappa coefficient (95%CI)
Male	0.229 (0.219 - 0.239)
Female	0.235 (0.223 - 0.247)
Without palliative care training	0.194 (0.172 - 0.216)
With palliative care training	0.238 (0.231 - 0.245)
Atheist	0.252 (0.225 - 0.280)
Intensivists with board certification or medical residency	0.238 (0.230 - 0.245)
Intensivists without board certification or medical residency	0.258 (0.149 - 0.368)
Works exclusively in a dedicated oncologic ICU	0.209 (0.165 - 0.254)
Works exclusively in general ICU	0.253 (0.226 - 0.280)
Works exclusively in public hospital ICU	0.163 (0.080 - 0.246)
Works exclusively in private hospital ICU	0.242 (0.225 - 0.260)

ICU - intensive care unit; 95%CI - confidence interval.
